# Relationship between Coffee, Tea, and Carbonated Beverages and Cardiovascular Risk Factors

**DOI:** 10.3390/nu15040934

**Published:** 2023-02-13

**Authors:** Hye-Ji An, Yejin Kim, Young-Gyun Seo

**Affiliations:** Department of Family Medicine, Hallym University Sacred Heart Hospital, Anyang 14068, Gyeonggi-do, Republic of Korea

**Keywords:** coffee, tea, carbonated beverage, hypertension, diabetes, dyslipidemia

## Abstract

We aimed to analyze the relationship between coffee, tea, and carbonated beverages and cardiovascular risk factors. We used data from the fourth to eighth Korea National Health and Nutrition Examination Surveys (2007–2016, 2019–2020). We categorized the frequency of intake into three groups (<1 time/week, 1 time/week to <1 time/day, and ≥1 time/day). Subsequently, logistic regression analyses by sex were performed to assess cardiovascular risk factors (hypertension (HTN), diabetes mellitus (DM), dyslipidemia (DL), or metabolic syndrome (MetS)) according to the frequency of coffee, tea, and carbonated beverage intake. For HTN, coffee intake showed an inverse relationship and tea intake showed a direct relationship. For DM, coffee intake showed an inverse relationship, and tea and carbonated beverage intake showed a direct relationship. For DL, coffee intake showed an inverse relationship, whereas tea intake demonstrated a direct relationship. In addition, carbonated beverage intake showed a direct relationship with MetS. Coffee intake showed an inverse relationship with HTN, DM, and DL. However, tea intake showed a direct relationship with HTN, DM, and DL, whereas carbonated beverage intake showed a direct relationship with DM and MetS.

## 1. Introduction

Coffee is the most popular beverage worldwide, with a 37% increase in per capita coffee consumption worldwide over the past 20 years [1], and 66% of Americans drink coffee daily [2]. In the case of Koreans, according to a survey on beverage and water intake of adults in 2019, coffee ranked first among beverages excluding water [3]. In addition, black and green teas are mainly consumed in West and East Asia, respectively [4], and tea consumption is increasing globally [5]. Regarding carbonated beverages, production in Korea increased by 13.9% in 2018 compared with 2014, accounting for the largest scale (45.5%) of the global beverage market [6].

Coffee contains micronutrients (magnesium, potassium, niacin, and vitamin E), caffeine, and chlorogenic acid [7]. Caffeine is involved in metabolic processes, such as increasing thermogenesis and metabolic rate or stimulating fat oxidation in peripheral tissues [8], and chlorogenic acid is expected to have the effect of preventing inflammation and oxidation. In addition, tea contains less caffeine, catechins, and other bioactive polyphenols (typically kaempferol) than coffee [9], and catechins and other polyphenols exhibit anti-tumor effects through interfering with cell division, activating programmed cell death, and inducing autophagocytosis [10].

Therefore, it was expected that tea and coffee would have health benefits. Many meta-analyses have demonstrated an association between tea or coffee and chronic metabolic diseases or cancer. A meta-analysis of patients with nonalcoholic fatty liver reported that coffee intake was inversely related to the liver fibrosis [11]. Consistent consumption of three to four cups of coffee per day was related to a reduced risk of all-cause and cardiovascular (CV) death, CV disease and cancer risk, and metabolic and liver disease compared to none at all [12]. In addition, two meta-analysis studies found high coffee intake to be inversely related to liver [13] and prostate [14] cancer, respectively. Another meta-analysis reported that tea intake was inversely related to all-cause, CV, and cancer deaths [15].

Thus, these studies support a trend toward increased consumption of coffee and tea worldwide. However, controversies still exist, as studies have shown that coffee intake is not significantly related to cancer death [16] or that tea consumption negatively affects disease [17]. In addition, there is a clinical study that cafestol, a diterpene contained in coffee, increases plasma triglycerides and low-density lipoprotein cholesterol [18]. However, various positive anti-inflammatory, anti-carcinogenic, and anti-diabetic effects of cafestol have also been reported [19].

Carbonated beverages contain various additives for flavor or food color, including artificial sweeteners for a sweet taste. Previous reviews have demonstrated that artificial sweeteners are associated with cardiometabolic risk [20,21]. Furthermore, beverages made with artificial sweeteners induce weight gain by reducing satiety [22], rapidly increasing blood sugar [23], and increasing the morbidity and mortality of CV disease [24]. In addition, although carbonated beverages also contain caffeine, the amount is not as high as that in coffee [25], and most caffeine intake in the United States population is driven by coffee and tea, while carbonated beverages make a small contribution to total caffeine intake [26].

CV disease is the leading cause of death, and obesity, metabolic syndrome (MetS), dyslipidemia (DL), diabetes mellitus (DM), and hypertension (HTN) are risk factors [27]. It is also associated with lifestyle factors such as lack of physical activity, excessive consumption of saturated fats, and tobacco and alcohol use [28].

Therefore, the aim of this study was to analyze the relationship between coffee, tea, and carbonated beverages and CV risk factors, including MetS, DL, DM, and HTN, using the data from the Korea National Health and Nutrition Examination Surveys (KNHANES).

## 2. Materials and Methods

### 2.1. Study Design and Participants

The KNHANES has been performed by the Korea Disease Control and Prevention Agency (KDCA) since 1998 to produce representative statistics on the health and nutritional status of Koreans. 75,160 participants aged 19 years or older were selected from the fourth to eighth surveys (2007–2016, 2019–2020; the food intake frequency survey was stopped from 2017 to 2018 to review the survey method). Among them, 32,547 were excluded according to the following exclusion criteria: missing test results or survey records; inadequate water intake (≥90 g/kg of body weight); inadequate nutritional intake (>5000 or <500 kcal/day); inadequate fasting time before test sampling (<8 or >24 h); renal dysfunction (estimated glomerular filtration rate <30); and a history of diagnosed cancer. Consequently, data from 42,613 participants (17,311 men and 25,302 women) comprised the final dataset.

All procedures were approved by the Ethics Committee of the KDCA (approval numbers 2018-01-03-2C-A, 2018-01-03-C-A, 2013-12EXP-03-5C, 2013-07CON-03-4C, 2012-01EXP-01-2C, 2011-02CON-06-C, 2010-02CON-21-C, 2009-01CON-03-2C, 2008-04EXP-01-C, and 2007-02CON-04-P) and were conducted in accordance with the ethical standards laid down in the 1964 Declaration of Helsinki and its later amendments. All KNHANES participants signed informed consent forms. The KNHANES data were made publicly available.

### 2.2. Coffee, Tea, and Carbonated Beverages Intake

Information on the frequency of coffee, tea, and carbonated beverage intake over the past year was obtained using the questionnaire. It consisted of the following 10 categories in 2007–2011: almost no intake, 6–11 times per year ((8.5/52.1)/week), 1 time per month ((1/4.3)/week), 2–3 times per month ((2.5/4.3)/week), 1 time per week (1/week), 2–3 times per week (2.5/week), 4–6 times per week (5/week), 1 time per day (1/day), 2 times per day (2/day), and 3 times per day (3/day). It consisted of the following nine categories in 2012–2016: almost no drinking, 1 time per month ((1/4.3)/week), 2–3 times per month ((2.5/4.3)/week), 1 time per week (1/week), 2–4 times per week (3/week), 5–6 times per week (5.5/week), 1 time per day (1/day), 2 times per day (2/day), and 3 times per day (3/day). It consisted of the following seven categories in 2019–2020: <1 time per week (0.5/week), 1 time per week (1/week), 2–4 times per week (3/week), 5–6 times per week (5.5/week), 1 time per day (1/day), 2 times per day (2/day), and 3 times per day (3/day). We categorized the frequency of intake as follows: group 1 (<1/week), group 2 (1/week to <1/day), and group 3 (≥1/day).

### 2.3. Other Variables

We used the following variables: age, daily nutritional intake (total energy, carbohydrate, protein, and fat intake), average monthly household income, education (≤elementary school, middle school, high school, or ≥college), smoking (no, past, or current smoker), alcohol drinking frequency (<1 or ≥1/month), walking (<30 or ≥30 min/day, 5 days/week), body mass index (BMI; <25 or ≥25 kg/m^2^), menopause status, and comorbidities (doctor-diagnosed HTN, DM, or DL).

Based on the 2001 National Cholesterol Education Program/Adult Treatment Panel III [29] and the 2005 American Heart Association/National Heart, Lung, and Blood Institute [30] criteria, we defined MetS when three or more of the following five factors were satisfied: (1) waist circumference ≥85 cm for women or ≥90 cm for men (Korean cutoff for abdominal obesity [31]); (2) serum triglyceride level ≥150 mg/dL or under treatment with drugs for DL; (3) serum high-density lipoprotein cholesterol level <50 mg/dL for women or <40 mg/dL for men; (4) blood pressure (BP) ≥130/85 mm Hg or under treatment with drugs for high BP; and (5) fasting plasma glucose level ≥100 mg/dL or under treatment with drugs for high glucose levels.

### 2.4. Statistical Analysis

We used STATA version 14.0 (StataCorp., College Station, TX, USA) for statistical analysis, and *p* < 0.05 was set as the statistical significance level. The KNHANES was conducted using a two-stage stratified cluster sampling method. Therefore, we assigned weights to the stratified data in our analysis.

Linear regression analyses and χ^2^ tests were used to analyze and compare the participants’ various characteristics according to sex and group. Logistic regression analyses by sex were used to assess CV risk factors (HTN, DM, DL, or MetS) according to coffee, tea, and carbonated beverage intake frequency. Adjusted odds ratios (ORs) were derived after controlling for potential confounding variables, such as age, daily nutritional intake (total and fat), average monthly household income, education, smoking, alcohol drinking, walking, BMI status, the frequency of intake of coffee, tea, and carbonated beverages, and menopausal status (only in women). In addition, the proportion of age groups according to the frequency of intake of coffee, tea, and carbonated beverages was derived. As a sensitivity analysis, logistic regression analyses were also conducted for participants 20 to less than 60 years.

## 3. Results

### 3.1. General Characteristics

Table S1 shows the participants’ general characteristics according to their sex. The average age of the 42,613 participants was 41.87 years, and 40.62% were men. The proportion of men who drink coffee, tea, and carbonated beverages once a day or more was higher compared to women. Total energy intake, protein and fat intake, and average monthly household income were higher, and carbohydrate intake was lower in men compared to women. Furthermore, the proportion of highly educated participants (≥college), current smokers, alcohol drinkers (≥1/month), participants walking more than 30 min per day, 5 days a week, participants with BMI ≥ 25 kg/m^2^, and participants with HTN, DM, and MetS were higher in men compared to women.

Tables S2–S7 show the participants’ general characteristics according to their coffee, tea, and carbonated beverage intake frequencies. In coffee, the proportion of men with HTN and DM was higher in group 1 (<1/week) than in groups 2 (1/week to <1/day) and 3 (≥1/day). The proportion of men with DL and MetS was higher in group 3 (≥1/day) than in groups 1 (<1/week) and 2 (1/week to <1/day) (Table S2). The proportion of women with HTN, DM, DL, and MetS was higher in group 1 (<1/week) than in groups 2 (1/week to <1/day) and 3 (≥1/day) (Table S3).

In tea, the proportion of men with HTN, DM, DL, and MetS was higher in group 3 (≥1/day) than in groups 1 (<1/week) and 2 (1/week to <1/day) (Table S4). The proportion of women with HTN, DM, and DL was higher in group 3 (≥1/day) than in groups 1 (<1/week) and 2 (1/week to <1/day). The proportion of women with MetS was higher in group 1 (<1/week) than in groups 2 (1/week to <1/day) and 3 (≥1/day) (Table S5).

In carbonated beverages, the proportion of men (Table S6) and women (Table S7) with HTN, DM, DL, and MetS was higher in group 1 (<1/week) than in groups 2 (1/week to <1/day) and 3 (≥1/day).

### 3.2. Cardiovascular Risk Factors by the Frequency of Intake of Coffee, Tea, and Carbonated Beverages

The multivariable logistic regression analysis results for HTN according to the frequency of coffee, tea, and carbonated beverage intake after adjusting for potential confounding variables are shown in Table 1. For coffee consumption in men, groups 2 (OR, 0.66; 95% CI, 0.49–0.90) and 3 (OR, 0.66; 95% CI, 0.52–0.84) had lower adjusted odds of doctor-diagnosed HTN than group 1. For tea consumption in men, groups 2 (OR, 1.46; 95% CI, 1.20–1.77) and 3 (OR, 2.60; 95% CI, 2.02–3.34) had higher adjusted odds of doctor-diagnosed HTN than group 1. For tea consumption in women, groups 2 (OR, 1.58; 95% CI, 1.30–1.92) and 3 (OR, 3.08; 95% CI, 2.21–4.30) had higher adjusted odds of doctor-diagnosed HTN than group 1.

The multivariable logistic regression analysis results for DM according to the frequency of coffee, tea, and carbonated beverage intake after adjusting for potential confounding variables are shown in Table 2. For coffee intake in men, group 3 (OR, 0.64; 95% CI, 0.47–0.87) had lower adjusted odds of doctor-diagnosed DM than group 1. For tea consumption in men, groups 2 (OR, 1.84; 95% CI, 1.40–2.42) and 3 (OR, 3.62; 95% CI, 2.64–4.96) had higher adjusted odds of doctor-diagnosed DM than group 1. For coffee consumption in women, groups 2 (OR 0.66; 95% CI, 0.50–0.88) and 3 (OR, 0.56; 95% CI, 0.44–0.71) had lower adjusted odds of doctor-diagnosed DM than group 1. For tea consumption in women, groups 2 (OR, 1.54; 95% CI, 1.19–1.99) and 3 (OR, 3.16; 95% CI, 2.16–4.62) had higher adjusted odds of doctor-diagnosed DM than group 1. For carbonated beverages in women, group 3 (OR, 4.29; 95% CI, 1.27–14.46) had higher adjusted odds of doctor-diagnosed DM than group 1.

The multivariable logistic regression analysis results for DL according to the frequency of coffee, tea, and carbonated beverage intake after adjusting for potential confounding variables are shown in Table 3. For coffee consumption in men, groups 2 (OR, 0.63; 95% CI, 0.46–0.85) and 3 (OR, 0.65; 95% CI, 0.50–0.84) had lower adjusted odds of doctor-diagnosed DL than group 1. For tea consumption in men, groups 2 (OR, 1.55; 95% CI, 1.26–1.92) and 3 (OR, 2.56; 95% CI, 1.93–3.41) had higher adjusted odds of doctor-diagnosed DL than group 1. For tea consumption in women, groups 2 (OR, 1.43; 95% CI, 1.18–1.73) and 3 (OR, 2.20; 95% CI, 1.60–3.01) had higher adjusted odds of doctor-diagnosed DL than group 1.

The multivariable logistic regression analysis results for MetS according to the frequency of coffee, tea, and carbonated beverage intake after adjusting for potential confounding variables are shown in Table 4. For carbonated beverages in women, groups 2 (OR, 1.19; 95% CI, 1.03–1.37) and 3 (OR, 2.10; 95% CI, 1.35–3.28) had higher adjusted odds of MetS than group 1.

### 3.3. Cardiovascular Risk Factors for Participants Aged 20 to Less than 60 Years

Although there were differences by age, most of them drank more coffee than other beverages, and the age group with a high percentage by category of intake pattern was limited to those in their 20s to 50s (Table S8); therefore, only this age group was reanalyzed.

For HTN, results that were similar to those of all age groups were obtained (Table S9).

For DM, results generally similar to those of all age groups were obtained, except for carbonated beverages in women, which provided non-significant results (Table S10).

For DL, results that were similar to those of all age groups were obtained (Table S11).

For MetS, results generally similar to those of all age groups were obtained, except for coffee in women, which provided statistically significant results (group 3: OR, 0.82; 95% CI, 0.72–0.95) (Table S12). 

### 3.4. Cardiovascular Risk Factors When the Frequency of Intake Is Categorized into Four Groups

The median frequency of intake was 1 time per day (1/day) (inter-quartile range [IQR], 1 time per week (1/week)–2 times per day (2/day)) for coffee, 6–11 times per year (8.5/52.1)/week (IQR, 0–1 time per week (1/week)) for tea, and 1 time per month (1/4.3)/week (IQR, 0–1 time per week (1/week)) for carbonated beverages. Therefore, we further categorized the frequency of coffee, tea, and carbonated beverages intake as follows: coffee group 1 (<1/week), coffee group 2 (1/week to <1/day), coffee group 3 (1/day to <2/day), and coffee group 4 (≥2/day); tea group 1 (<(8.5/52.1)/week), tea group 2 ((8.5/52.1)/week) to <1/week), tea group 3 (1/week to <1/day), and tea group 4 (≥1/day); carbonated beverages group 1 (<(1/4.3)/week), carbonated beverages group 2 ((1/4.3)/week) to <1/week), carbonated beverages group 3 (1/week to <1/day), and carbonated beverages group 4 (≥1/day).

For HTN, results generally similar to those of the three groups were obtained, except for coffee group 3 in men, which provided non-significant results, and coffee group 4 and tea group 2 in women, which provided statistically significant results (coffee group 4 in women: OR, 0.77; 95% CI, 0.63–0.94; and tea group 2 in women: OR, 1.27; 95% CI, 1.04–1.57) (Table S13).

For DM, results generally similar to those of the three groups were obtained, except for coffee group 3 in both men and women, which provided non-significant results (Table S14).

For DL, results generally similar to those of three groups were obtained, except for coffee group 3 in men, which provided non-significant results, and tea group 2 in men and tea group 2 and carbonated beverages group 4 in women, which provided statistically significant results (tea group 2 in men: OR, 1.37; 95% CI, 1.07–1.75, and tea group 2 in women: OR, 1.28; 95% CI, 1.04–1.57, and carbonated beverages group 4 in women: OR, 2.72; 95% CI, 1.01–7.35) (Table S15).

For MetS, results generally similar to those of the three groups were obtained, except for coffee group 3 in women, which provided non-significant results, and carbonated beverages group 2 in women, which provided statistically significant results (carbonated beverages group 2 in women: OR, 1.19; 95% CI, 1.05–1.34) (Table S16).

## 4. Discussion

In this study, HTN and DL were related to low coffee and high tea consumption, respectively. DM was related to low coffee, high tea, and high carbonated beverage intake in women. MetS has been linked to a high intake of carbonated beverages in women. When the frequency of coffee intake was subdivided, coffee consumption between once and less than twice a day was not associated with cardiovascular risk factors.

The following points are in the same direction as the previous studies: (1) coffee intake is inversely related to the risk of HTN [32] and DM [33], and (2) beverages made with artificial sweeteners are related to the risk of DM [34]. (3) In addition, both sweetened and artificially sweetened beverages, which are also a type of carbonated beverage, are associated with MetS [35].

However, the following points are inconsistent with the previous studies: (1) tea consumption was able to reduce blood levels of fasting glucose in participants younger than 55 years old [36]. (2) both tea and coffee intake are less likely to develop MetS [11]. (3) Coffee may be related to the risk of DL. [37]

The following pathophysiology partially supports the findings of our study: (1) A prospective cohort study found that the pesticide residue (1,1,1-trichloro-2,2-bis(p-chlorophenyl) ethane) of oolong tea may have been involved in the association between long-term oolong tea intake and the DM risk [38]. (2) Chlorogenic acid, which is a major component of coffee, is involved in lipid metabolism, increases fat oxidation by upregulating peroxisome proliferator-activated receptor alpha (fenofibrate-like action), and reduces fatty acid synthesis through inhibiting 3-hydroxy-3-methylglutaryl-coenzyme A reductase and hepatic fatty acid synthase (statin-like action) [39]. However, further studies are needed to clarify these differences from previous studies.

Although meta-analyses of prospective studies have found that tea intake is related to a reduced risk of DM and MetS [11,36], a few cross-sectional studies have found that tea intake is related to a higher BMI and metabolic abnormalities [17,40]. This is consistent with our study, in that tea intake was related to higher odds of HTN, DM, and DL in both women and men. This might be explained by the reverse causality that participants with chronic diseases are more likely to care for their diets and receive nutritional counseling than those without chronic diseases. For example, in a Canadian population of 98,733 adolescents to the elderly, participants with DM, heart disease, or cancer were more inclined to select or avoid foods based on health considerations or food content than subjects without these conditions [41]. Similarly, in a study of 1399 Italians, participants with DM had a healthier diet than participants without DM in both food and beverage choices; patients with DM had more calories from vegetables, fruits, and eggs than those without DM and consumed less juice but more water [42]. Considering that tea is one of Asia’s most consumed beverages and its health benefits are well known, tea intake may increase when the participant’s health deteriorates. Another possible explanation is the presence of plastic substances in tea bags. Tea bags are mainly made of plastic, such as nylon [43], which enables the release of significant amounts of plastic-related particles [44]. Additionally, the migration of cyclic oligomers was observed in a polyamide-6 (nylon 6) tea bag in hot water [45]. A recent study detected that one plastic tea bag at brewing temperature discharged 11.6 billion micro- and 3.1 billion nano-plastics into a single cup, respectively [46]. Although the association between microplastics and nanoplastics and health risk is unclear, microplastics and nanoplastics can induce oxidative stress, inflammation [47], and carcinogenesis [48].

Our study found that individuals who consumed more carbonated beverages had a higher risk of DM and MetS than those who consumed fewer carbonated beverages. This can be explained by the main sources of carbonated beverages, fructose, and liquid carbohydrates, which increase the overall calorie intake due to decreased satiety and induce central obesity, insulin resistance, and CV disease [22,23,34]. In contrast, maintaining or reducing the consumption of carbonated beverages or replacing them with water, tea, or coffee was related to a reduced risk of DM [49]. Moreover, frequent consumption of carbonated beverages can be a proxy indicator of an unhealthy lifestyle, such as lower physical activity levels, a poor diet, and smoking [50,51]. Individuals who consumed carbonated beverages once a day or more had higher calorie intake, current smoking, and alcohol consumption than those who consumed less than once a week in our study. However, the association between the consumption of carbonated beverages, DM, and MetS was only statistically significant in women. This is consistent with a 10-year prospective study in Korean adults, where soft beverage consumption was associated with an increased incidence of MetS and elevated its components only in women [52]. Additionally, a recent cross-sectional study found that sugar-sweetened beverage intake is related to metabolic risk, particularly in women [53]. This sex difference may be explained by sex hormone levels. Estrogen is crucial for fundamental sex differences when lipids and carbohydrates are used as fuel sources [54]. Estrogen affects the renin-angiotensin system, which activates the transport of fat and increases triglyceride and total cholesterol levels, whereas androgens have the opposite effect in men [55]. This can cause the response to carbohydrate or fat intake to become more sensitive in women. Therefore, sex hormones may have contributed to the relationship between carbonated beverage intake and DM and MetS, particularly in women. 

In our study, a significant difference was found in the association between the frequency of coffee and tea consumption and the adjusted odds of doctor-diagnosed HTN, DM, and DL. However, no difference was observed in the relationship with MetS. Due to the nature of MetS, which is a cluster of three or more CV risk factors, coffee and tea consumption are insufficient to influence more than three components at once. Since many factors cause MetS, it is impossible to control them when conducting a study; therefore, it might be challenging to demonstrate our thoughts.

There were some limitations in this study. First, as this was a cross-sectional design, we could not evaluate the cause-and-effect relationship between beverages (coffee, tea, and carbonated beverages) and each disease (HTN, DM, DL, and MetS). Therefore, it is necessary to conduct a randomized controlled trial to confirm whether the same results can be obtained in clinical practice. Second, it did not reflect the diversity of the detailed types and ingredients of beverages. In the case of coffee, there are various types, and the content of components such as caffeine and chlorogenic acid differs depending on the type of coffee. There are also various types of tea, as well as the three main types of green tea, black tea, and oolong tea, according to the degree of fermentation, and each tea has a different type and amount of polyphenols. Therefore, studies should be performed to confirm the association between more subcategorized beverage types and diseases. Third, when collecting data, the format of the survey on dietary habits was changed in the middle (24-h recall survey in 2007–2017, open type; dietary survey in 2018–2019, categorical type). Therefore, there is a possibility of data inconsistency. Future studies need to consider these limitations.

## 5. Conclusions

In this study, coffee intake showed an inverse relationship with HTN, DM, and DL. However, tea intake showed a direct relationship with HTN, DM, and DL, and carbonated beverage intake showed a direct relationship with DM and MetS. Therefore, we expect to prevent metabolic diseases, such as HTN, DM, DL, and MetS, in adults and further reduce CV disease morbidity by improving the amount and type of beverage consumption.

## Figures and Tables

**Table 1:** Multivariable logistic regression for hypertension according to the frequency of intake of coffee, tea, and carbonated beverages.

	Crude	Model 1	Model 2	Model 3
Men				
Coffee intake				
<1 time/week	reference	reference	reference	reference
1 time/week~<1 time/day	0.53 (0.43–0.64)	0.77 (0.58–1.01)	0.74 (0.56–0.98)	0.66 (0.49–0.90)
≥1 time/day	1.11 (0.94–1.32)	0.70 (0.56–0.87)	0.67 (0.54–0.85)	0.66 (0.52–0.84)
Tea intake				
<1 time/week	reference	reference	reference	reference
1 time/week~<1 time/day	0.75 (0.65–0.87)	1.37 (1.13–1.66)	1.37 (1.13–1.66)	1.46 (1.20–1.77)
≥1 time/day	1.93 (1.58–2.35)	2.41 (1.90–3.08)	2.44 (1.91–3.12)	2.60 (2.02–3.34)
Carbonated beverage intake				
<1 time/week	reference	reference	reference	reference
1 time/week~<1 time/day	0.31 (0.27–0.36)	1.12 (0.92–1.36)	1.11 (0.91–1.35)	1.09 (0.89–1.33)
≥1 time/day	0.18 (0.10–0.31)	1.02 (0.52–1.97)	1.04 (0.53–2.02)	1.08 (0.55–2.11)
Women				
Coffee intake				
<1 time/week	reference	reference	reference	reference
1 time/week~<1 time/day	0.49 (0.42–0.57)	1.10 (0.88–1.38)	1.04 (0.83–1.30)	0.99 (0.78–1.26)
≥1 time/day	0.75 (0.66–0.85)	0.95 (0.80–1.12)	0.89 (0.75–1.06)	0.92 (0.77–1.11)
Tea intake				
<1 time/week	reference	reference	reference	reference
1 time/week~<1 time/day	0.68 (0.59–0.77)	1.41 (1.17–1.71)	1.40 (1.16–1.69)	1.58 (1.30–1.92)
≥1 time/day	1.47 (1.18–1.83)	2.79 (2.05–3.78)	2.77 (2.04–3.76)	3.08 (2.21–4.30)
Carbonated beverage intake				
<1 time/week	reference	reference	reference	reference
1 time/week~<1 time/day	0.24 (0.21–0.29)	1.30 (1.03–1.64)	1.25 (0.98–1.58)	1.11 (0.87–1.43)
≥1 time/day	0.36 (0.19–0.68)	2.66 (1.16–6.11)	2.43 (1.08–5.44)	2.31 (0.86–6.22)

The data are shown as odds ratios with 95% confidence intervals. Model 1: adjusted for age. Model 2: adjusted for age and frequency of coffee, tea, and carbonated beverage intake. Model 3: adjusted for age, the frequency of intake of coffee, tea, and carbonated beverages, daily nutritional intake (total and fat), income, education, smoking, alcohol drinking, walking, body mass index status, and menopausal status (only in women).

**Table 2:** Multivariable logistic regression for diabetes according to the frequency of intake of coffee, tea, and carbonated beverages.

	Crude	Model 1	Model 2	Model 3
Men				
Coffee intake				
<1 time/week	reference	reference	reference	reference
1 time/week~<1 time/day	0.54 (0.40–0.72)	0.83 (0.56–1.21)	0.84 (0.57–1.24)	0.89 (0.60–1.31)
≥1 time/day	0.92 (0.72–1.17)	0.58 (0.4–0.79)	0.59 (0.44–0.81)	0.64 (0.47–0.87)
Tea intake				
<1 time/week	reference	reference	reference	reference
1 time/week~<1 time/day	0.82 (0.67–1.00)	1.56 (1.22–2.00)	1.54 (1.20–1.98)	1.84 (1.40–2.42)
≥1 time/day	2.22 (1.74–2.84)	2.66 (1.98–3.56)	2.68 (2.00–3.59)	3.62 (2.64–4.96)
Carbonated beverage intake				
<1 time/week	reference	reference	reference	reference
1 time/week~<1 time/day	0.22 (0.18–0.28)	0.89 (0.68–1.17)	0.89 (0.68–1.16)	0.90 (0.68–1.19)
≥1 time/day	0.20 (0.10–0.42)	1.22 (0.53–2.82)	1.32 (0.57–3.02)	1.24 (0.52–2.97)
Women				
Coffee intake				
<1 time/week	reference	reference	reference	reference
1 time/week~<1 time/day	0.43 (0.35–0.53)	0.73 (0.56–0.94)	0.69 (0.53–0.90)	0.66 (0.5–0.88)
≥1 time/day	0.51 (0.43–0.61)	0.53 (0.42–0.66)	0.49 (0.40–0.62)	0.56 (0.44–0.71)
Tea intake				
<1 time/week	reference	reference	reference	reference
1 time/week~<1 time/day	0.69 (0.56–0.84)	1.28 (1.01–1.63)	1.30 (1.02–1.65)	1.54 (1.19–1.99)
≥1 time/day	1.53 (1.13–2.08)	2.72 (1.90–3.90)	2.87 (1.98–4.15)	3.16 (2.16–4.62)
Carbonated beverage intake				
<1 time/week	reference	reference	reference	reference
1 time/week~<1 time/day	0.26 (0.20–0.34)	1.31 (0.94–1.82)	1.33 (0.95–1.88)	1.17 (0.83–1.67)
≥1 time/day	0.76 (0.33–1.74)	5.88 (1.75–19.79)	5.58 (1.71–18.19)	4.29 (1.27–14.46)

The data are shown as odds ratios with 95% confidence intervals. Model 1: adjusted for age. Model 2: adjusted for age and frequency of coffee, tea, and carbonated beverage intake. Model 3: adjusted for age, the frequency of intake of coffee, tea, and carbonated beverages, daily nutritional intake (total and fat), income, education, smoking, alcohol drinking, walking, body mass index status, and menopausal status (only in women).

**Table 3:** Multivariable logistic regression for dyslipidemia according to the frequency of intake of coffee, tea, and carbonated beverages.

	Crude	Model 1	Model 2	Model 3
Men				
Coffee intake				
<1 time/week	reference	reference	reference	reference
1 time/week~<1 time/day	0.52 (0.39–0.68)	0.70 (0.52–0.95)	0.67 (0.50–0.91)	0.63 (0.46–0.85)
≥1 time/day	1.10 (0.89–1.36)	0.73 (0.57–0.92)	0.70 (0.55–0.90)	0.65 (0.50–0.84)
Tea intake				
<1 time/week	reference	reference	reference	reference
1 time/week~<1 time/day	0.87 (0.73–1.04)	1.39 (1.13–1.69)	1.41 (1.15–1.72)	1.55 (1.26–1.92)
≥1 time/day	2.24 (1.76–2.85)	2.50 (1.89–3.31)	2.51 (1.89–3.33)	2.56 (1.93–3.41)
Carbonated beverage intake				
<1 time/week	reference	reference	reference	reference
1 time/week~<1 time/day	0.39 (0.33–0.47)	1.09 (0.88–1.36)	1.08 (0.87–1.35)	1.10 (0.88–1.38)
≥1 time/day	0.24 (0.13–0.43)	0.90 (0.47–1.70)	0.94 (0.49–1.81)	1.01 (0.52–1.94)
Women				
Coffee intake				
<1 time/week	reference	reference	reference	reference
1 time/week~<1 time/day	0.55 (0.45–0.65)	0.93 (0.74–1.17)	0.90 (0.71–1.13)	0.88 (0.70–1.11)
≥1 time/day	0.90 (0.78–1.05)	0.92 (0.76–1.10)	0.88 (0.73–1.07)	0.93 (0.77–1.13)
Tea intake				
<1 time/week	reference	reference	reference	reference
1 time/week~<1 time/day	0.74 (0.63–0.86)	1.34 (1.11–1.61)	1.35 (1.12–1.63)	1.43 (1.18–1.73)
≥1 time/day	1.35 (1.06–1.74)	2.21 (1.62–3.01)	2.23 (1.63–3.04)	2.20 (1.60–3.01)
Carbonated beverage intake				
<1 time/week	reference	reference	reference	reference
1 time/week~<1 time/day	0.23 (0.19–0.28)	0.98 (0.76–1.25)	0.95 (0.74–1.22)	0.89 (0.69–1.15)
≥1 time/day	0.41 (0.20–0.81)	3.16 (1.02–9.76)	3.09 (1.04–9.17)	2.58 (0.94–7.11)

The data are shown as odds ratios with 95% confidence intervals. Model 1: adjusted for age. Model 2: adjusted for age and frequency of coffee, tea, and carbonated beverage intake. Model 3: adjusted for age, the frequency of intake of coffee, tea, and carbonated beverages, daily nutritional intake (total and fat), income, education, smoking, alcohol drinking, walking, body mass index status, and menopausal status (only in women).

**Table 4:** Multivariable logistic regression for metabolic syndrome according to the frequency of intake of coffee, tea, and carbonated beverages.

	Crude	Model 1	Model 2	Model 3
Men				
Coffee intake				
<1 time/week	reference	reference	reference	reference
1 time/week~<1 time/day	0.96 (0.82–1.13)	1.06 (0.90–1.26)	1.05 (0.89–1.25)	1.00 (0.82–1.21)
≥1 time/day	1.55 (1.37–1.75)	1.34 (1.18–1.52)	1.32 (1.16–1.50)	1.10 (0.95–1.28)
Tea intake				
<1 time/week	reference	reference	reference	reference
1 time/week~<1 time/day	0.91 (0.82–1.01)	1.08 (0.97–1.21)	1.10 (0.99–1.23)	0.99 (0.87–1.13)
≥1 time/day	1.22 (1.05–1.40)	1.28 (1.10–1.48)	1.24 (1.07–1.44)	1.05 (0.88–1.24)
Carbonated beverage intake				
<1 time/week	reference	reference	reference	reference
1 time/week~<1 time/day	0.65 (0.60–0.72)	1.01 (0.91–1.11)	0.99 (0.89–1.09)	1.03 (0.92–1.16)
≥1 time/day	0.55 (0.42–0.73)	0.98 (0.74–1.31)	0.96 (0.72–1.28)	0.96 (0.68–1.34)
Women				
Coffee intake				
<1 time/week	reference	reference	reference	reference
1 time/week~<1 time/day	0.74 (0.64–0.84)	1.03 (0.89–1.19)	1.03 (0.89–1.19)	1.01 (0.86–1.19)
≥1 time/day	0.90 (0.81–1.00)	0.97 (0.87–1.08)	0.95 (0.85–1.07)	0.91 (0.80–1.04)
Tea intake				
<1 time/week	reference	reference	reference	reference
1 time/week~<1 time/day	0.66 (0.60–0.74)	0.94 (0.83–1.05)	0.92 (0.82–1.03)	0.98 (0.86–1.12)
≥1 time/day	0.90 (0.77–1.06)	1.15 (0.97–1.36)	1.15 (0.97–1.36)	1.15 (0.95–1.38)
Carbonated beverage intake				
<1 time/week	reference	reference	reference	reference
1 time/week~<1 time/day	0.55 (0.49–0.62)	1.32 (1.16–1.50)	1.32 (1.16–1.50)	1.19 (1.03–1.37)
≥1 time/day	0.63 (0.42–0.95)	1.82 (1.14–2.91)	1.80 (1.13–2.89)	2.10 (1.35–3.28)

The data are shown as odds ratios with 95% confidence intervals. Model 1: adjusted for age. Model 2: adjusted for age and frequency of coffee, tea, and carbonated beverage intake. Model 3: adjusted for age, the frequency of intake of coffee, tea, and carbonated beverages, daily nutritional intake (total and fat), income, education, smoking, alcohol drinking, walking, body mass index status, and menopausal status (only in women).

## Data Availability

The data presented in this study are publicly available in the Korea National Health and Nutrition Examination Survey database at https://knhanes.kdca.go.kr/knhanes/sub03/sub03_02_05.do (accessed on 11 January 2023) and https://knhanes.kdca.go.kr/knhanes/eng/index.do (accessed on 11 January 2023).

## Supplementary Materials

The following is available online at https://www.mdpi.com/article/10.3390/nu15040934/s1. Table S1: General characteristics of the participants by sex; Table S2: General characteristics of men by the frequency of intake of coffee; Table S3: General characteristics of women by the frequency of intake of coffee; Table S4: General characteristics of men by the frequency of intake of tea; Table S5: General characteristics of women by the frequency of intake of tea; Table S6: General characteristics of men by the frequency of intake of carbonated beverages; Table S7: General characteristics of women by the frequency of intake of carbonated beverages; Table S8: Proportion of age groups by the frequency of intake of coffee, tea, and carbonated beverages; Table S9: Multivariable logistic regression for hypertension according to the frequency of intake of coffee, tea, and carbonated beverages (age ≥20 and <60 years); Table S10: Multivariable logistic regression for diabetes according to the frequency of intake of coffee, tea, and carbonated beverages (age ≥20 and <60 years); Table S11: Multivariable logistic regression for dyslipidemia according to the frequency of intake of coffee, tea, and carbonated beverages (age ≥20 and <60 years); Table S12: Multivariable logistic regression for metabolic syndrome according to the frequency of intake of coffee, tea, and carbonated beverages (age ≥20 and <60 years); Table S13: Multivariable logistic regression for hypertension when the frequency of intake is categorized into four groups; Table S14: Multivariable logistic regression for diabetes when the frequency of intake is categorized into four groups; Table S15: Multivariable logistic regression for dyslipidemia when the frequency of intake is categorized into four groups; Table S16: Multivariable logistic regression for metabolic syndrome when the frequency of intake is categorized into four groups.
