# Supplementary material for: Relationship between Coffee, Tea, and Carbonated Beverages and Cardiovascular Risk Factors
Source: Nutrients. 2023 Feb 13;15(4):934. doi: 10.3390/nu15040934 (PMC9966641; doi:10.3390/nu15040934)
Supplement: Supplementary file 1 [file nutrients-15-00934-s001.zip › nutrients-2185669-supplementary.pdf]

Table S1. General characteristics of the participants by sex

	Total (n = 42613)	Men (n = 17311)	Women (n = 25302)	p value
Age, years	41.87±0.13	41.34±0.15	42.39±0.15	<0.001
Coffee intake				<0.001
<1 time/week	7750 (18.19)	2575 (14.87)	5175 (20.45)	
1 time/week ~ <1 time/day	7605 (17.85)	2948 (17.03)	4657 (18.41)	
≥1 time/day	27258 (63.97)	11788 (68.10)	15470 (61.14)	
Tea intake				<0.001
<1 time/week	28952 (67.94)	11270 (65.10)	17682 (69.88)	
1 time/week ~ <1 time/day	9318 (21.87)	4000 (23.11)	5318 (21.02)	
≥1 time/day	4343 (10.19)	2041 (11.79)	2302 (9.10)	
Carbonated beverage intake				<0.001
<1 time/week	31995 (75.08)	11486 (66.35)	20509 (81.06)	
1 time/week ~ <1 time/day	9876 (23.18)	5364 (30.99)	4512 (17.83)	
≥1 time/day	742 (1.74)	461 (2.66)	281 (1.11)	
Nutritional intake				
Total energy intake, kcal/day	2,043.14±6.02	2,371.05±8.89	1,717.84±5.57	<0.001
Carbohydrates, % of energy	63.46±0.11	60.88±0.15	66.02±0.12	<0.001
Protein, % of energy	14.36±0.03	14.48±0.04	14.25±0.04	<0.001
Fat, % of energy	19.12±0.07	19.27±0.09	18.98±0.09	0.007
Average monthly household income, 10,000 KRW	381.10±3.28	385.12±3.81	377.09±3.42	0.009
Education				<0.001
≤Elementary school	7896 (20.24)	2271 (14.70)	5625 (23.88)	
Middle school	3942 (10.11)	1666 (10.78)	2276 (9.66)	
High school	14184 (36.36)	5834 (37.75)	8350 (35.45)	
≥College	12985 (33.29)	5683 (36.77)	7302 (31.00)	
Smoking				<0.001
None	25581 (64.94)	3959 (25.33)	21622 (91.00)	
Past	6301 (16.00)	5353 (34.25)	948 (3.99)	
Current	7508 (19.06)	6318 (40.42)	1190 (5.01)	
Alcohol drinking				<0.001
<1 time/month	17950 (45.63)	4096 (26.24)	13854 (58.39)	
≥1 time/month	21386 (54.37)	11512 (73.76)	9874 (41.61)	
Walking				<0.001
<30 minute * 5 days/week	22786 (58.51)	8636 (55.93)	14150 (60.19)	
≥30 minute * 5 days/week	16161 (41.49)	6804 (44.07)	9357 (39.81)	
Body mass index				<0.001
<25 kg/m^2	27414 (68.51)	10012 (62.83)	17402 (72.27)	

≥25 kg/m^2	12599 (31.49)	5923 (37.17)	6676 (27.73)	
Hypertension	6773 (37.25)	2934 (39.73)	3839 (35.56)	<0.001
Diabetes	2494 (16.93)	1213 (20.19)	1281 (14.69)	<0.001
Dyslipidemia	3570 (23.57)	1361 (22.79)	2209 (24.08)	0.067
Metabolic syndrome	8706 (21.70)	4123 (25.79)	4583 (18.99)	<0.001

KRW: Korea republic won.

Data are presented as mean ± standard error for continuous variables (linear regression) and as numbers (%) for categorical variables ($\chi 2$ test).

Tables S2. General characteristics of men by the frequency of intake of coffee

	<1 time/week (n = 2575)	1 time/week ~ <1 time/day (n = 2948)	≥1 time/day (n = 11788)	p value
Age, years	38.18±0.39	35.59±0.30	43.74±0.17	<0.001
Tea intake				<0.001
<1 time/week	1894 (73.55)	1561 (52.95)	7815 (66.30)	
1 time/week ~ <1 time/day	471 (18.29)	1207 (40.94)	2322 (19.70)	
≥1 time/day	210 (8.16)	180 (6.11)	1651 (14.01)	
Carbonated beverage intake				<0.001
<1 time/week	1876 (72.85)	1649 (55.94)	7961 (67.53)	
1 time/week ~ <1 time/day	638 (24.78)	1222 (41.45)	3504 (29.73)	
≥1 time/day	61 (2.37)	77 (2.61)	323 (2.74)	
Nutritional intake				
Total energy intake, kcal/day	2,270.07±21.94	2,343.02±19.32	2,403.05±10.31	<0.001
Carbohydrates, % of energy	61.13±0.39	59.15±0.33	61.32±0.17	0.024
Protein, % of energy	14.58±0.11	15.03±0.11	14.30±0.05	<0.001
Fat, % of energy	19.20±0.25	20.75±0.22	18.85±0.10	<0.001
Average monthly household income, 10,000 KRW	346.35±7.46	404.09±7.90	388.75±4.06	<0.001
Education				<0.001
≤Elementary school	426 (18.30)	353 (13.28)	1492 (14.25)	
Middle school	223 (9.58)	212 (7.97)	1231 (11.76)	
High school	940 (40.38)	1185 (44.57)	3709 (35.44)	
≥College	739 (31.74)	909 (34.19)	4035 (38.55)	
Smoking				<0.001
None	968 (41.31)	998 (37.20)	1993 (18.79)	
Past	788 (33.63)	853 (31.79)	3712 (35.01)	
Current	587 (25.05)	832 (31.01)	4899 (46.20)	
Alcohol drinking				<0.001
<1 time/month	757 (32.31)	648 (24.17)	2691 (25.43)	
≥1 time/month	1586 (67.69)	2033 (75.83)	7893 (74.57)	
Walking				<0.001
<30 minute * 5 days/week	1263 (54.28)	1374 (51.69)	5999 (57.38)	
≥30 minute * 5 days/week	1064 (45.72)	1284 (48.31)	4456 (42.62)	
Body mass index				<0.001
<25 kg/m^2	1633 (68.50)	1736 (63.59)	6643 (61.39)	
≥25 kg/m^2	751 (31.50)	994 (36.41)	4178 (38.61)	
Hypertension	454 (43.53)	433 (29.76)	2047 (41.89)	<0.001
Diabetes	197 (23.68)	186 (14.86)	830 (21.15)	<0.001
Dyslipidemia	193 (24.16)	185 (15.02)	983 (24.94)	<0.001

Metabolic syndrome	534 (22.34)	587 (21.41)	3002 (27.66)	<0.001

KRW: Korea republic won.

Data are presented as mean ± standard error for continuous variables (linear regression) and as numbers (%) for categorical variables ($\chi 2$ test).

Tables S3. General characteristics of women by the frequency of intake of coffee

	<1 time/week (n = 5175)	1 time/week ~ <1 time/day (n = 4657)	≥1 time/day (n = 15470)	p value
Age, years	42.48±0.32	37.59±0.28	43.95±0.16	<0.001
Tea intake				<0.001
<1 time/week	4193 (81.02)	2791 (59.93)	10698 (69.15)	
1 time/week ~ <1 time/day	735 (14.20)	1642 (35.26)	2941 (19.01)	
≥1 time/day	247 (4.77)	224 (4.81)	1831 (11.84)	
Carbonated beverage intake				<0.001
<1 time/week	4434 (85.68)	3416 (73.35)	12659 (81.83)	
1 time/week ~ <1 time/day	690 (13.33)	1189 (25.53)	2633 (17.02)	
≥1 time/day	51 (0.99)	52 (1.12)	178 (1.15)	
Nutritional intake				
Total energy intake, kcal/day	1,707.91±12.63	1,703.48±12.05	1,725.88±6.64	0.113
Carbohydrates, % of energy	67.58±0.24	64.21±0.25	66.11±0.14	0.002
Protein, % of energy	14.14±0.07	14.47±0.08	14.21±0.05	0.974
Fat, % of energy	17.88±0.18	20.34±0.19	18.89±0.10	0.007
Average monthly household income, 10,000 KRW	326.74±5.06	399.68±5.82	386.12±3.91	<0.001
Education				<0.001
≤Elementary school	1567 (32.52)	969 (22.30)	3089 (21.47)	
Middle school	426 (8.84)	343 (7.89)	1507 (10.47)	
High school	1524 (31.63)	1649 (37.94)	5177 (35.98)	
≥College	1301 (27.00)	1385 (31.87)	4616 (32.08)	
Smoking				<0.001
None	4501 (92.69)	4024 (91.77)	13097 (90.21)	
Past	194 (4.00)	171 (3.90)	583 (4.02)	
Current	161 (3.32)	190 (4.33)	839 (5.78)	
Alcohol drinking				<0.001
<1 time/month	3510 (72.45)	2524 (57.61)	7820 (53.92)	
≥1 time/month	1335 (27.55)	1857 (42.39)	6682 (46.08)	
Walking				0.026
<30 minute * 5 days/week	2857 (59.38)	2554 (58.86)	8739 (60.87)	
≥30 minute * 5 days/week	1954 (40.62)	1785 (41.14)	5618 (39.13)	
Body mass index				<0.001
<25 kg/m^2	3644 (74.02)	3274 (73.96)	10484 (71.18)	
≥25 kg/m^2	1279 (25.98)	1153 (26.04)	4244 (28.82)	
Hypertension	995 (44.80)	650 (29.78)	2194 (34.32)	<0.001
Diabetes	417 (24.46)	229 (12.41)	635 (12.28)	<0.001
Dyslipidemia	502 (29.24)	358 (18.70)	1349 (24.34)	<0.001

Metabolic syndrome	1082 (21.92)	795 (17.91)	2706 (18.34)	<0.001
Menopause	2501 (50.79)	1674 (37.93)	5765 (39.44)	<0.001

KRW: Korea republic won.

Data are presented as mean ± standard error for continuous variables (linear regression) and as numbers (%) for categorical variables ($\chi 2$ test).

Tables S4. General characteristics of men by the frequency of intake of tea

	<1 time/week (n = 11270)	1 time/week ~ <1 time/day (n = 4000)	≥1 time/day (n = 2041)	p value
Age, years	42.49±0.19	38.10±0.25	41.94±0.32	<0.001
Coffee intake				<0.001
<1 time/week	1894 (16.81)	471 (11.77)	210 (10.29)	
1 time/week ~ <1 time/day	1561 (13.85)	1207 (30.18)	180 (8.82)	
≥1 time/day	7815 (69.34)	2322 (58.05)	1651 (80.89)	
Carbonated beverage intake				<0.001
<1 time/week	7902 (70.12)	2258 (56.45)	1326 (64.97)	
1 time/week ~ <1 time/day	3064 (27.19)	1640 (41.00)	660 (32.34)	
≥1 time/day	304 (2.70)	102 (2.55)	55 (2.69)	
Nutritional intake				
Total energy intake, kcal/day	2,374.48±10.59	2,374.13±16.82	2,346.37±24.97	0.381
Carbohydrates, % of energy	61.53±0.18	59.33±0.28	60.66±0.42	<0.001
Protein, % of energy	14.16±0.05	15.07±0.09	14.96±0.13	<0.001
Fat, % of energy	18.83±0.11	20.50±0.18	19.02±0.24	<0.001
Average monthly household income, 10,000 KRW	365.77±4.33	423.55±6.78	408.14±7.71	<0.001
Education				<0.001
≤Elementary school	1887 (18.76)	275 (7.66)	109 (6.04)	
Middle school	1260 (12.53)	275 (7.66)	131 (7.26)	
High school	3781 (37.59)	1445 (40.24)	608 (33.68)	
≥College	3130 (31.12)	1596 (44.44)	957 (53.02)	
Smoking				<0.001
None	2363 (23.24)	1163 (32.01)	433 (23.65)	
Past	3519 (34.62)	1141 (31.41)	693 (37.85)	
Current	4284 (42.14)	1329 (36.58)	705 (38.50)	
Alcohol drinking				<0.001
<1 time/month	2859 (28.16)	844 (23.25)	393 (21.51)	
≥1 time/month	7292 (71.84)	2786 (76.75)	1434 (78.49)	
Walking				0.007
<30 minute * 5 days/week	5714 (56.85)	1936 (54.05)	986 (54.57)	
≥30 minute * 5 days/week	4337 (43.15)	1646 (45.95)	821 (45.43)	
Body mass index				<0.001
<25 kg/m^2	6779 (65.17)	2186 (59.29)	1047 (56.72)	
≥25 kg/m^2	3623 (34.83)	1501 (40.71)	799 (43.28)	
Hypertension	2035 (40.15)	558 (33.14)	341 (53.96)	<0.001
Diabetes	807 (19.63)	242 (17.10)	164 (34.10)	<0.001
Dyslipidemia	886 (21.95)	284 (19.80)	191 (38.05)	<0.001

Metabolic syndrome	2680 (25.69)	906 (24.49)	537 (28.96)	0.001

KRW: Korea republic won.

Data are presented as mean ± standard error for continuous variables (linear regression) and as numbers (%) for categorical variables (χ2 test).

Tables S5. General characteristics of women by the frequency of intake of tea

	<1 time/week (n = 17682)	1 time/week ~ <1 time/day (n = 5318)	≥1 time/day (n = 2302)	p value
Age, years	43.84±0.18	38.42±0.23	41.30±0.35	<0.001
Coffee intake				<0.001
<1 time/week	4193 (23.71)	735 (13.82)	247 (10.73)	
1 time/week ~ <1 time/day	2791 (15.78)	1642 (30.88)	224 (9.73)	
≥1 time/day	10698 (60.50)	2941 (55.30)	1831 (79.54)	
Carbonated beverage intake				<0.001
<1 time/week	14755 (83.45)	3941 (74.11)	1813 (78.76)	
1 time/week ~ <1 time/day	2750 (15.55)	1310 (24.63)	452 (19.64)	
≥1 time/day	177 (1.00)	67 (1.26)	37 (1.61)	
Nutritional intake				
Total energy intake, kcal/day	1,716.56±6.45	1,718.55±11.70	1,725.50±18.85	0.656
Carbohydrates, % of energy	66.73±0.14	64.27±0.23	65.11±0.33	<0.001
Protein, % of energy	14.02±0.04	14.65±0.07	14.96±0.12	<0.001
Fat, % of energy	18.44±0.10	20.39±0.17	19.53±0.24	<0.001
Average monthly household income, 10,000 KRW	360.49±3.69	423.96±6.16	385.86±7.70	<0.001
Education				<0.001
≤Elementary school	4658 (28.23)	676 (13.63)	291 (13.91)	
Middle school	1603 (9.72)	450 (9.07)	223 (10.66)	
High school	5445 (33.00)	2027 (40.86)	878 (41.97)	
≥College	4794 (29.05)	1808 (36.44)	700 (33.46)	
Smoking				0.029
None	15109 (90.76)	4590 (91.71)	1923 (91.27)	
Past	706 (4.24)	174 (3.48)	68 (3.23)	
Current	833 (5.00)	241 (4.82)	116 (5.51)	
Alcohol drinking				<0.001
<1 time/month	10168 (61.14)	2654 (53.13)	1032 (49.10)	
≥1 time/month	6463 (38.86)	2341 (46.87)	1070 (50.90)	
Walking				<0.001
<30 minute * 5 days/week	10151 (61.62)	2852 (57.58)	1147 (55.14)	
≥30 minute * 5 days/week	6323 (38.38)	2101 (42.42)	933 (44.86)	
Body mass index				<0.001
<25 kg/m^2	12094 (71.58)	3780 (74.94)	1528 (71.44)	
≥25 kg/m^2	4801 (28.42)	1264 (25.06)	611 (28.56)	
Hypertension	2982 (36.08)	602 (30.73)	255 (44.58)	<0.001
Diabetes	988 (14.77)	205 (12.67)	88 (21.26)	<0.001
Dyslipidemia	1672 (23.99)	380 (21.99)	157 (32.98)	<0.001

Metabolic syndrome	3442 (20.32)	774 (15.31)	367 (17.13)	<0.001
Menopause	7618 (45.52)	1620 (32.05)	702 (32.41)	<0.001

KRW: Korea republic won.

Data are presented as mean ± standard error for continuous variables (linear regression) and as numbers (%) for categorical variables (χ2 test).

Tables S6. General characteristics of men by the frequency of intake of carbonated beverages

	<1 time/week (n = 11486)	1 time/week ~ <1 time/day (n = 5364)	≥1 time/day (n = 461)	p value
Age, years	46.20±0.17	34.38±0.20	30.64±0.48	<0.001
Coffee intake				<0.001
<1 time/week	1876 (16.33)	638 (11.89)	61 (13.23)	
1 time/week ~ <1 time/day	1649 (14.36)	1222 (22.78)	77 (16.70)	
≥1 time/day	7961 (69.31)	3504 (65.32)	323 (70.07)	
Tea intake				<0.001
<1 time/week	7902 (68.80)	3064 (57.12)	304 (65.94)	
1 time/week ~ <1 time/day	2258 (19.66)	1640 (30.57)	102 (22.13)	
≥1 time/day	1326 (11.54)	660 (12.30)	55 (11.93)	
Nutritional intake				
Total energy intake, kcal/day	2,317.01±10.43	2,443.38±14.77	2,544.90±56.14	<0.001
Carbohydrates, % of energy	62.20±0.19	58.86±0.22	59.52±0.73	<0.001
Protein, % of energy	14.31±0.05	14.77±0.07	14.42±0.27	<0.001
Fat, % of energy	17.75±0.10	21.44±0.15	22.53±0.52	<0.001
Average monthly household income, 10,000 KRW	375.90±4.18	397.93±5.77	408.68±18.39	<0.001
Education				<0.001
≤Elementary school	1943 (18.74)	310 (6.60)	18 (4.59)	
Middle school	1332 (12.85)	315 (6.71)	19 (4.85)	
High school	3521 (33.96)	2101 (44.76)	212 (54.08)	
≥College	3572 (34.45)	1968 (41.93)	143 (36.48)	
Smoking				<0.001
None	2389 (22.80)	1458 (30.69)	112 (27.86)	
Past	4167 (39.77)	1137 (23.94)	49 (12.19)	
Current	3922 (37.43)	2155 (45.37)	241 (59.95)	
Alcohol drinking				<0.001
<1 time/month	2861 (27.34)	1141 (24.06)	94 (23.38)	
≥1 time/month	7603 (72.66)	3601 (75.94)	308 (76.62)	
Walking				0.303
<30 minute * 5 days/week	5838 (56.32)	2590 (55.28)	208 (53.47)	
≥30 minute * 5 days/week	4528 (43.68)	2095 (44.72)	181 (46.53)	
Body mass index				0.051
<25 kg/m^2	6776 (63.48)	2985 (61.56)	251 (60.92)	
≥25 kg/m^2	3898 (36.52)	1864 (38.44)	161 (39.08)	
Hypertension	2443 (48.78)	471 (21.51)	20 (10.70)	<0.001
Diabetes	1057 (26.95)	146 (7.67)	10 (5.49)	<0.001
Dyslipidemia	1097 (28.70)	247 (12.54)	17 (9.34)	<0.001

Metabolic syndrome	3008 (28.09)	1034 (21.25)	81 (19.66)	<0.001

KRW: Korea republic won.

Data are presented as mean ± standard error for continuous variables (linear regression) and as numbers (%) for categorical variables ($\chi2$ test).

Tables S7. General characteristics of women by the frequency of intake of carbonated beverages

	<1 time/week (n = 20509)	1 time/week ~ <1 time/day (n = 4512)	≥1 time/day (n = 281)	p value
Age, years	45.32±0.16	32.65±0.21	30.56±0.78	<0.001
Coffee intake				<0.001
<1 time/week	4434 (21.62)	690 (15.29)	51 (18.15)	
1 time/week ~ <1 time/day	3416 (16.66)	1189 (26.35)	52 (18.51)	
≥1 time/day	12659 (61.72)	2633 (58.36)	178 (63.35)	
Tea intake				<0.001
<1 time/week	14755 (71.94)	2750 (60.95)	177 (62.99)	
1 time/week ~ <1 time/day	3941 (19.22)	1310 (29.03)	67 (23.84)	
≥1 time/day	1813 (8.84)	452 (10.02)	37 (13.17)	
Nutritional intake				
Total energy intake, kcal/day	1,694.68±6.10	1,791.10±12.73	1,863.23±50.61	<0.001
Carbohydrates, % of energy	67.26±0.13	61.93±0.23	61.12±0.96	<0.001
Protein, % of energy	14.21±0.04	14.43±0.08	13.72±0.34	0.182
Fat, % of energy	18.01±0.10	22.28±0.18	21.84±0.68	<0.001
Average monthly household income, 10,000 KRW	370.45±3.62	399.79±5.79	394.37±23.39	<0.001
Education				<0.001
≤Elementary school	5179 (27.03)	427 (10.29)	19 (7.72)	
Middle school	2007 (10.48)	257 (6.19)	12 (4.88)	
High school	6433 (33.58)	1809 (43.60)	108 (43.90)	
≥College	5539 (28.91)	1656 (39.91)	107 (43.50)	
Smoking				<0.001
None	17789 (92.01)	3640 (87.10)	193 (77.82)	
Past	714 (3.69)	215 (5.14)	19 (7.66)	
Current	830 (4.29)	324 (7.75)	36 (14.52)	
Alcohol drinking				<0.001
<1 time/month	11819 (61.21)	1930 (46.25)	105 (42.51)	
≥1 time/month	7489 (38.79)	2243 (53.75)	142 (57.49)	
Walking				0.081
<30 minute * 5 days/week	11556 (60.41)	2463 (59.58)	131 (53.91)	
≥30 minute * 5 days/week	7574 (39.59)	1671 (40.42)	112 (46.09)	
Body mass index				0.001
<25 kg/m^2	14064 (71.76)	3147 (74.50)	191 (75.20)	
≥25 kg/m^2	5536 (28.24)	1077 (25.50)	63 (24.80)	
Hypertension	3529 (39.89)	291 (15.86)	19 (16.67)	<0.001
Diabetes	1183 (17.01)	89 (5.36)	9 (8.65)	<0.001
Dyslipidemia	2039 (27.67)	156 (9.19)	14 (13.08)	<0.001

Metabolic syndrome	4001 (20.37)	546 (12.90)	36 (14.12)	<0.001
Menopause	9137 (46.94)	768 (18.13)	35 (13.89)	<0.001

KRW: Korea republic won.

Data are presented as mean ± standard error for continuous variables (linear regression) and as numbers (%) for categorical variables ($\chi 2$ test).

Tables S8. Proportion of age groups by the frequency of intake of coffee, tea, and carbonated beverages

Coffee intake	Tea intake	Carbonated beverage intake	10s	20s	30s	40s	50s	60s	70s	≥80s	Total
<1 time/week	<1 time/week	<1 time/week	105 (2.10)	670 (13.40)	827 (16.54)	583 (11.66)	991 (19.82)	958 (19.16)	689 (13.78)	177 (3.54)	5000 (100.00)
1 time/week ~ <1 time/day	<1 time/week	<1 time/week	37 (1.18)	357 (11.37)	541 (17.22)	466 (14.84)	685 (21.81)	622 (19.80)	360 (11.46)	73 (2.32)	3141 (100.00)
<1 time/week	1 time/week ~ <1 time/day	<1 time/week	7 (0.74)	133 (14.07)	160 (16.93)	170 (17.99)	201 (21.27)	177 (18.73)	87 (9.21)	10 (1.06)	945 (100.00)
<1 time/week	<1 time/week	1 time/week ~ <1 time/day	106 (10.59)	457 (45.65)	220 (21.98)	75 (7.49)	53 (5.29)	45 (4.50)	33 (3.30)	12 (1.20)	1001 (100.00)
1 time/week ~ <1 time/day	1 time/week ~ <1 time/day	<1 time/week	26 (1.55)	401 (23.93)	282 (16.83)	299 (17.84)	308 (18.38)	251 (14.98)	98 (5.85)	11 (0.66)	1676 (100.00)
1 time/week ~ <1 time/day	<1 time/week	1 time/week ~ <1 time/day	99 (8.66)	519 (45.41)	256 (22.40)	117 (10.24)	79 (6.91)	41 (3.59)	27 (2.36)	5 (0.44)	1143 (100.00)
<1 time/week	1 time/week ~ <1 time/day	1 time/week ~ <1 time/day	17 (6.94)	101 (41.22)	62 (25.31)	25 (10.20)	22 (8.98)	10 (4.08)	8 (3.27)	0 (0.00)	245 (100.00)
1 time/week ~ <1 time/day	1 time/week ~ <1 time/day	1 time/week ~ <1 time/day	82 (7.31)	753 (67.17)	134 (11.95)	69 (6.16)	44 (3.93)	28 (2.50)	9 (0.80)	2 (0.18)	1121 (100.00)
≥1 time/day	<1 time/week	<1 time/week	14 (0.10)	668 (4.60)	2783 (19.17)	3463 (23.86)	3668 (25.27)	2564 (17.66)	1159 (7.98)	197 (1.36)	14516 (100.00)
<1 time/week	≥1 time/day	<1 time/week	2 (0.55)	52 (14.25)	52 (14.25)	100 (27.40)	86 (23.56)	59 (16.16)	14 (3.84)	0 (0.00)	365 (100.00)
<1 time/week	<1 time/week	≥1 time/day	14 (16.28)	48 (55.81)	13 (15.12)	4 (4.65)	4 (4.65)	3 (3.49)	0 (0.00)	0 (0.00)	86 (100.00)
≥1 time/day	1 time/week ~ <1 time/day	<1 time/week	3 (0.08)	266 (7.43)	740 (20.68)	1096 (30.63)	866 (24.20)	463 (12.94)	123 (3.44)	21 (0.59)	3578 (100.00)
≥1 time/day	<1 time/week	1 time/week ~ <1 time/day	30 (0.82)	690 (18.80)	1264 (34.44)	817 (22.26)	487 (13.27)	254 (6.92)	108 (2.94)	20 (0.54)	3670 (100.00)
1 time/week ~ <1 time/day	≥1 time/day	<1 time/week	2 (0.81)	60 (24.19)	34 (13.71)	51 (20.56)	56 (22.58)	33 (13.31)	12 (4.84)	0 (0.00)	248 (100.00)
<1 time/week	≥1 time/day	1 time/week ~ <1 time/day	1 (1.22)	35 (42.68)	17 (20.73)	14 (17.07)	7 (8.54)	7 (8.54)	0 (0.00)	1 (1.22)	82 (100.00)
1 time/week ~ <1 time/day	<1 time/week	≥1 time/day	3 (4.41)	43 (63.24)	16 (23.53)	4 (5.88)	1 (1.47)	1 (1.47)	0 (0.00)	0 (0.00)	68 (100.00)
<1 time/week	1 time/week ~ <1 time/day	≥1 time/day	1 (6.25)	5 (31.25)	7 (43.75)	1 (6.25)	1 (6.25)	0 (0.00)	1 (6.25)	0 (0.00)	16 (100.00)

≥1 time/day	1 time/week ~ <1 time/day	1 time/week ~ <1 time/day	13 (0.82)	472 (29.80)	496 (31.31)	346 (21.84)	170 (10.73)	63 (3.98)	21 (1.33)	3 (0.19)	1584 (100.00)
1 time/week ~ <1 time/day	≥1 time/day	1 time/week ~ <1 time/day	13 (8.84)	84 (57.14)	28 (19.05)	13 (8.84)	5 (3.40)	2 (1.36)	2 (1.36)	0 (0.00)	147 (100.00)
1 time/week ~ <1 time/day	1 time/week ~ <1 time/day	≥1 time/day	7 (13.46)	37 (71.15)	7 (13.46)	0 (0.00)	1 (1.92)	0 (0.00)	0 (0.00)	0 (0.00)	52 (100.00)
≥1 time/day	≥1 time/day	<1 time/week	3 (0.12)	154 (6.10)	517 (20.47)	785 (31.08)	640 (25.34)	302 (11.96)	105 (4.16)	20 (0.79)	2526 (100.00)
≥1 time/day	<1 time/week	≥1 time/day	8 (2.45)	102 (31.19)	108 (33.03)	57 (17.43)	33 (10.09)	13 (3.98)	4 (1.22)	2 (0.61)	327 (100.00)
<1 time/week	≥1 time/day	≥1 time/day	0 (0.00)	7 (70.00)	2 (20.00)	1 (10.00)	0 (0.00)	0 (0.00)	0 (0.00)	0 (0.00)	10 (100.00)
≥1 time/day	≥1 time/day	1 time/week ~ <1 time/day	6 (0.68)	188 (21.29)	324 (36.69)	221 (25.03)	101 (11.44)	30 (3.40)	11 (1.25)	2 (0.23)	883 (100.00)
≥1 time/day	1 time/week ~ <1 time/day	≥1 time/day	6 (5.94)	47 (46.53)	28 (27.72)	15 (14.85)	3 (2.97)	2 (1.98)	0 (0.00)	0 (0.00)	101 (100.00)
1 time/week ~ <1 time/day	≥1 time/day	≥1 time/day	1 (11.11)	7 (77.78)	1 (11.11)	0 (0.00)	0 (0.00)	0 (0.00)	0 (0.00)	0 (0.00)	9 (100.00)
≥1 time/day	≥1 time/day	≥1 time/day	1 (1.37)	31 (42.47)	21 (28.77)	13 (17.81)	5 (6.85)	1 (1.37)	1 (1.37)	0 (0.00)	73 (100.00)

Table S9. Multivariable logistic regression for hypertension according to the frequency of intake of coffee, tea, and carbonated beverages (age ≥20 and <60 years)

	Crude	Model 1	Model 2	Model 3
Men				
Coffee intake				
<1 time/week	reference	reference	reference	reference
1 time/week ~ <1 time/day	0.58 (0.46 to 0.74)	0.85 (0.65 to 1.12)	0.83 (0.63 to 1.09)	0.73 (0.55 to 0.98)
≥1 time/day	1.13 (0.93 to 1.36)	0.78 (0.63 to 0.97)	0.75 (0.61 to 0.94)	0.70 (0.56 to 0.89)
Tea intake				
<1 time/week	reference	reference	reference	reference
1 time/week ~ <1 time/day	0.86 (0.74 to 1.01)	1.35 (1.14 to 1.61)	1.35 (1.13 to 1.61)	1.38 (1.14 to 1.66)
≥1 time/day	2.48 (2.04 to 3.02)	2.72 (2.18 to 3.39)	2.74 (2.19 to 3.42)	2.85 (2.25 to 3.60)
Carbonated beverage intake				
<1 time/week	reference	reference	reference	reference
1 time/week ~ <1 time/day	0.41 (0.36 to 0.48)	1.09 (0.92 to 1.29)	1.07 (0.90 to 1.28)	1.05 (0.88 to 1.27)
≥1 time/day	0.23 (0.14 to 0.38)	0.81 (0.46 to 1.42)	0.84 (0.47 to 1.47)	0.85 (0.48 to 1.51)
Women				
Coffee intake				
<1 time/week	reference	reference	reference	reference
1 time/week ~ <1 time/day	0.65 (0.53 to 0.79)	1.04 (0.83 to 1.31)	0.98 (0.78 to 1.24)	0.95 (0.74 to 1.22)
≥1 time/day	1.00 (0.86 to 1.17)	0.96 (0.80 to 1.14)	0.91 (0.76 to 1.08)	0.95 (0.79 to 1.16)
Tea intake				
<1 time/week	reference	reference	reference	reference
1 time/week ~ <1 time/day	0.99 (0.85 to 1.14)	1.52 (1.28 to 1.80)	1.51 (1.27 to 1.79)	1.69 (1.41 to 2.03)
≥1 time/day	2.03 (1.64 to 2.51)	2.43 (1.88 to 3.15)	2.44 (1.88 to 3.16)	2.64 (2.00 to 3.48)
Carbonated beverage intake				
<1 time/week	reference	reference	reference	reference
1 time/week ~ <1 time/day	0.40 (0.33 to 0.47)	1.27 (1.03 to 1.57)	1.23 (1.00 to 1.52)	1.09 (0.87 to 1.36)
≥1 time/day	0.40 (0.21 to 0.78)	1.37 (0.62 to 3.02)	1.34 (0.61 to 2.94)	1.18 (0.51 to 2.73)

Data are presented as odds ratio (95% confidence interval).

Model 1: Adjusted for age.

Model 2: Adjusted for age, the frequency of intake of coffee, tea, and carbonated beverages.

Model 3: Adjusted for age, the frequency of intake of coffee, tea, and carbonated beverages, daily nutritional intake (total and fat), average monthly household income, education level, smoking, alcohol drinking, walking, body mass index status, and menopause status (only in women).

Table S10. Multivariable logistic regression for diabetes according to the frequency of intake of coffee, tea, and carbonated beverages (age ≥20 and <60 years)

	Crude	Model 1	Model 2	Model 3
Men				
Coffee intake				
<1 time/week	reference	reference	reference	reference
1 time/week ~ <1 time/day	0.63 (0.45 to 0.87)	0.99 (0.69 to 1.43)	1.02 (0.71 to 1.48)	1.00 (0.67 to 1.47)
≥1 time/day	1.00 (0.77 to 1.31)	0.72 (0.54 to 0.96)	0.72 (0.54 to 0.96)	0.69 (0.50 to 0.95)
Tea intake				
<1 time/week	reference	reference	reference	reference
1 time/week ~ <1 time/day	0.99 (0.80 to 1.24)	1.62 (1.28 to 2.06)	1.62 (1.28 to 2.07)	1.90 (1.46 to 2.46)
≥1 time/day	2.68 (2.06 to 3.48)	2.97 (2.23 to 3.96)	3.04 (2.28 to 4.06)	4.21 (3.09 to 5.72)
Carbonated beverage intake				
<1 time/week	reference	reference	reference	reference
1 time/week ~ <1 time/day	0.29 (0.23 to 0.37)	0.85 (0.66 to 1.11)	0.84 (0.65 to 1.10)	0.83 (0.63 to 1.10)
≥1 time/day	0.23 (0.11 to 0.49)	0.91 (0.41 to 2.04)	0.98 (0.44 to 2.20)	0.79 (0.34 to 1.83)
Women				
Coffee intake				
<1 time/week	reference	reference	reference	reference
1 time/week ~ <1 time/day	0.46 (0.35 to 0.62)	0.66 (0.48 to 0.89)	0.62 (0.46 to 0.85)	0.62 (0.45 to 0.87)
≥1 time/day	0.58 (0.47 to 0.72)	0.52 (0.41 to 0.65)	0.49 (0.39 to 0.62)	0.58 (0.46 to 0.75)
Tea intake				
<1 time/week	reference	reference	reference	reference
1 time/week ~ <1 time/day	0.96 (0.76 to 1.23)	1.40 (1.08 to 1.80)	1.42 (1.10 to 1.83)	1.62 (1.24 to 2.12)
≥1 time/day	2.18 (1.57 to 3.02)	2.59 (1.83 to 3.66)	2.77 (1.95 to 3.93)	2.90 (2.00 to 4.20)
Carbonated beverage intake				
<1 time/week	reference	reference	reference	reference
1 time/week ~ <1 time/day	0.46 (0.35 to 0.61)	1.17 (0.86 to 1.58)	1.19 (0.88 to 1.62)	1.00 (0.72 to 1.38)
≥1 time/day	0.92 (0.42 to 1.99)	2.61 (1.12 to 6.07)	2.68 (1.16 to 6.21)	1.97 (0.82 to 4.75)

Data are presented as odds ratio (95% confidence interval).

Model 1: Adjusted for age.

Model 2: Adjusted for age, the frequency of intake of coffee, tea, and carbonated beverages.

Model 3: Adjusted for age, the frequency of intake of coffee, tea, and carbonated beverages, daily nutritional intake (total and fat), average monthly household income, education level, smoking, alcohol drinking, walking, body mass index status, and menopause status (only in women).

Table S11. Multivariable logistic regression for dyslipidemia according to the frequency of intake of coffee, tea, and carbonated beverages (age ≥20 and <60 years)

	Crude	Model 1	Model 2	Model 3
Men				
Coffee intake				
<1 time/week	reference	reference	reference	reference
1 time/week ~ <1 time/day	0.59 (0.44 to 0.78)	0.81 (0.60 to 1.10)	0.78 (0.57 to 1.06)	0.73 (0.53 to 1.001)
≥1 time/day	1.12 (0.89 to 1.40)	0.82 (0.65 to 1.04)	0.80 (0.62 to 1.02)	0.73 (0.57 to 0.95)
Tea intake				
<1 time/week	reference	reference	reference	reference
1 time/week ~ <1 time/day	0.94 (0.78 to 1.12)	1.39 (1.14 to 1.68)	1.40 (1.15 to 1.71)	1.49 (1.22 to 1.83)
≥1 time/day	2.37 (1.89 to 2.97)	2.51 (1.98 to 3.20)	2.52 (1.98 to 3.21)	2.61 (2.03 to 3.36)
Carbonated beverage intake				
<1 time/week	reference	reference	reference	reference
1 time/week ~ <1 time/day	0.46 (0.39 to 0.54)	1.04 (0.86 to 1.26)	1.03 (0.84 to 1.25)	1.02 (0.83 to 1.25)
≥1 time/day	0.32 (0.19 to 0.56)	0.96 (0.54 to 1.71)	1.01 (0.56 to 1.81)	1.03 (0.57 to 1.86)
Women				
Coffee intake				
<1 time/week	reference	reference	reference	reference
1 time/week ~ <1 time/day	0.55 (0.44 to 0.69)	0.82 (0.64 to 1.05)	0.79 (0.61 to 1.01)	0.78 (0.60 to 1.01)
≥1 time/day	0.98 (0.83 to 1.16)	0.90 (0.75 to 1.09)	0.87 (0.72 to 1.05)	0.95 (0.78 to 1.16)
Tea intake				
<1 time/week	reference	reference	reference	reference
1 time/week ~ <1 time/day	0.98 (0.84 to 1.16)	1.52 (1.26 to 1.82)	1.53 (1.27 to 1.84)	1.61 (1.33 to 1.95)
≥1 time/day	1.88 (1.49 to 2.39)	2.36 (1.79 to 3.11)	2.37 (1.80 to 3.13)	2.35 (1.76 to 3.14)
Carbonated beverage intake				
<1 time/week	reference	reference	reference	reference
1 time/week ~ <1 time/day	0.36 (0.29 to 0.44)	1.09 (0.87 to 1.38)	1.07 (0.85 to 1.35)	0.95 (0.75 to 1.21)
≥1 time/day	0.61 (0.33 to 1.12)	2.28 (1.09 to 4.77)	2.31 (1.11 to 4.80)	1.81 (0.85 to 3.85)

Data are presented as odds ratio (95% confidence interval).

Model 1: Adjusted for age.

Model 2: Adjusted for age, the frequency of intake of coffee, tea, and carbonated beverages.

Model 3: Adjusted for age, the frequency of intake of coffee, tea, and carbonated beverages, daily nutritional intake (total and fat), average monthly household income, education level, smoking, alcohol drinking, walking, body mass index status, and menopause status (only in women).

Table S12. Multivariable logistic regression for metabolic syndrome according to the frequency of intake of coffee, tea, and carbonated beverages (age ≥20 and <60 years)

	Crude	Model 1	Model 2	Model 3
Men				
Coffee intake				
<1 time/week	reference	reference	reference	reference
1 time/week ~ <1 time/day	0.87 (0.74 to 1.02)	0.96 (0.82 to 1.14)	0.94 (0.80 to 1.12)	0.88 (0.72 to 1.06)
≥1 time/day	1.28 (1.13 to 1.46)	1.09 (0.96 to 1.25)	1.08 (0.94 to 1.23)	0.92 (0.79 to 1.08)
Tea intake				
<1 time/week	reference	reference	reference	reference
1 time/week ~ <1 time/day	0.96 (0.87 to 1.06)	1.10 (0.99 to 1.21)	1.11 (1.001 to 1.23)	1.02 (0.90 to 1.15)
≥1 time/day	1.20 (1.06 to 1.36)	1.19 (1.05 to 1.35)	1.18 (1.04 to 1.34)	1.04 (0.90 to 1.20)
Carbonated beverage intake				
<1 time/week	reference	reference	reference	reference
1 time/week ~ <1 time/day	0.70 (0.64 to 0.77)	1.04 (0.94 to 1.15)	1.03 (0.93 to 1.14)	1.03 (0.92 to 1.16)
≥1 time/day	0.70 (0.54 to 0.90)	1.22 (0.94 to 1.59)	1.21 (0.93 to 1.58)	1.10 (0.81 to 1.49)
Women				
Coffee intake				
<1 time/week	reference	reference	reference	reference
1 time/week ~ <1 time/day	0.93 (0.80 to 1.07)	1.09 (0.93 to 1.27)	1.08 (0.93 to 1.26)	1.02 (0.85 to 1.21)
≥1 time/day	1.05 (0.93 to 1.17)	0.94 (0.83 to 1.06)	0.92 (0.82 to 1.04)	0.82 (0.72 to 0.95)
Tea intake				
<1 time/week	reference	reference	reference	reference
1 time/week ~ <1 time/day	0.80 (0.72 to 0.89)	0.93 (0.83 to 1.04)	0.91 (0.81 to 1.01)	0.97 (0.85 to 1.10)
≥1 time/day	0.97 (0.84 to 1.12)	1.03 (0.89 to 1.20)	1.04 (0.90 to 1.21)	1.06 (0.90 to 1.26)
Carbonated beverage intake				
<1 time/week	reference	reference	reference	reference
1 time/week ~ <1 time/day	0.74 (0.66 to 0.83)	1.42 (1.25 to 1.61)	1.43 (1.26 to 1.62)	1.20 (1.04 to 1.38)
≥1 time/day	0.88 (0.59 to 1.31)	2.06 (1.35 to 3.16)	2.08 (1.36 to 3.18)	2.05 (1.28 to 3.29)

Data are presented as odds ratio (95% confidence interval).

Model 1: Adjusted for age.

Model 2: Adjusted for age, the frequency of intake of coffee, tea, and carbonated beverages.

Model 3: Adjusted for age, the frequency of intake of coffee, tea, and carbonated beverages, daily nutritional intake (total and fat), average monthly household income, education level, smoking, alcohol drinking, walking, body mass index status, and menopause status (only in women).

Table S13. Multivariable logistic regression for hypertension when the frequency of intake is categorized into four groups

	Crude	Model 1	Model 2	Model 3
Men				
Coffee intake				
<1 time/week	reference	reference	reference	reference
1 time/week ~ <1 time/day	0.53 (0.43 to 0.64)	0.77 (0.58 to 1.01)	0.74 (0.56 to 0.98)	0.66 (0.49 to 0.90)
1 time/day ~ <2 times/day	1.42 (1.16 to 1.75)	1.04 (0.80 to 1.35)	0.99 (0.75 to 1.30)	0.99 (0.74 to 1.32)
≥2 times/day	1.02 (0.86 to 1.21)	0.62 (0.49 to 0.77)	0.60 (0.48 to 0.76)	0.57 (0.44 to 0.73)
Green tea intake				
<6–11 times/year	reference	reference	reference	reference
6–11 times/year ~ <1 time/week	1.01 (0.85 to 1.19)	1.15 (0.94 to 1.41)	1.11 (0.90 to 1.36)	1.14 (0.92 to 1.42)
1 time/week ~ <1 time/day	0.75 (0.65 to 0.87)	1.42 (1.16 to 1.72)	1.37 (1.12 to 1.67)	1.47 (1.20 to 1.80)
≥1 time/day	1.93 (1.58 to 2.36)	2.49 (1.94 to 3.19)	2.48 (1.93 to 3.20)	2.66 (2.05 to 3.45)
Carbonated beverage intake				
<1 time/month	reference	reference	reference	reference
1 time/month ~ <1 time/week	0.45 (0.39 to 0.52)	1.02 (0.85 to 1.22)	1.04 (0.86 to 1.25)	1.07 (0.88 to 1.30)
1 time/week ~ <1 time/day	0.23 (0.20 to 0.27)	1.13 (0.91 to 1.40)	1.12 (0.91 to 1.39)	1.11 (0.89 to 1.39)
≥1 time/day	0.13 (0.078 to 0.23)	1.02 (0.53 to 1.99)	1.02 (0.51 to 2.03)	1.08 (0.55 to 2.12)
Women				
Coffee intake				
<1 time/week	reference	reference	reference	reference
1 time/week ~ <1 time/day	0.49 (0.42 to 0.57)	1.10 (0.87 to 1.37)	1.02 (0.82 to 1.27)	0.98 (0.77 to 1.24)
1 time/day ~ <2 times/day	1.01 (0.87 to 1.16)	1.24 (1.02 to 1.51)	1.15 (0.94 to 1.40)	1.18 (0.95 to 1.46)
≥2 times/day	0.59 (0.52 to 0.68)	0.79 (0.66 to 0.95)	0.74 (0.61 to 0.89)	0.77 (0.63 to 0.94)
Green tea intake				
<6–11 times/year	reference	reference	reference	reference
6–11 times/year ~ <1 time/week	1.01 (0.88 to 1.16)	1.20 (0.99 to 1.45)	1.18 (0.98 to 1.42)	1.27 (1.04 to 1.57)
1 time/week ~ <1 time/day	0.68 (0.59 to 0.77)	1.46 (1.20 to 1.77)	1.44 (1.18 to 1.75)	1.65 (1.35 to 2.01)
≥1 time/day	1.47 (1.18 to 1.83)	2.88 (2.12 to 3.92)	2.83 (2.08 to 3.85)	3.20 (2.28 to 4.49)
Carbonated beverage intake				
<1 time/month	reference	reference	reference	reference
1 time/month ~ <1 time/week	0.41 (0.36 to 0.46)	1.16 (0.97 to 1.40)	1.19 (0.99 to 1.43)	1.14 (0.94 to 1.38)
1 time/week ~ <1 time/day	0.19 (0.16 to 0.22)	1.35 (1.06 to 1.72)	1.32 (1.03 to 1.69)	1.16 (0.89 to 1.50)
≥1 time/day	0.28 (0.15 to 0.53)	2.77 (1.20 to 6.41)	2.53 (1.13 to 1.18)	2.36 (0.87 to 6.38)

Data are presented as odds ratio (95% confidence interval).

Model 1: Adjusted for age.

Model 2: Adjusted for age, the frequency of intake of coffee, tea, and carbonated beverages.

Model 3: Adjusted for age, the frequency of intake of coffee, tea, and carbonated beverages, daily nutritional intake (total and fat), average monthly household income, education level, smoking, alcohol drinking, walking, body mass index status, and menopause status (only in women).

Table S14. Multivariable logistic regression for diabetes when the frequency of intake is categorized into four groups

	Crude	Model 1	Model 2	Model 3
Men				
Coffee intake				
<1 time/week	reference	reference	reference	reference
1 time/week ~ <1 time/day	0.54 (0.40 to 0.72)	0.82 (0.56 to 1.21)	0.84 (0.57 to 1.24)	0.89 (0.60 to 1.31)
1 time/day ~ <2 times/day	1.24 (0.94 to 1.62)	0.94 (0.66 to 1.34)	0.92 (0.65 to 1.31)	1.01 (0.71 to 1.45)
≥2 times/day	0.82 (0.63 to 1.05)	0.50 (0.36 to 0.68)	0.51 (0.37 to 0.70)	0.54 (0.39 to 0.74)
Green tea intake				
<6–11 times/year	reference	reference	reference	reference
6–11 times/year ~ <1 time/week	0.97 (0.77 to 1.23)	1.09 (0.84 to 1.42)	1.04 (0.79 to 1.36)	1.22 (0.91 to 1.63)
1 time/week ~ <1 time/day	0.82 (0.66 to 1.002)	1.59 (1.23 to 2.06)	1.52 (1.17 to 1.97)	1.88 (1.40 to 2.52)
≥1 time/day	2.21 (1.71 to 2.84)	2.71 (2.00 to 3.66)	2.65 (1.96 to 3.60)	3.72 (2.68 to 5.16)
Carbonated beverage intake				
<1 time/month	reference	reference	reference	reference
1 time/month ~ <1 time/week	0.42 (0.35 to 0.52)	1.07 (0.85 to 1.36)	1.11 (0.87 to 1.41)	1.12 (0.87 to 1.45)
1 time/week ~ <1 time/day	0.17 (0.13 to 0.21)	0.91 (0.69 to 1.20)	0.93 (0.71 to 1.23)	0.93 (0.69 to 1.25)
≥1 time/day	0.15 (0.073 to 0.32)	1.25 (0.54 to 2.91)	1.23 (0.53 to 2.88)	1.19 (0.49 to 2.86)
Women				
Coffee intake				
<1 time/week	reference	reference	reference	reference
1 time/week ~ <1 time/day	0.43 (0.35 to 0.53)	0.72 (0.56 to 0.94)	0.68 (0.52 to 0.89)	0.65 (0.49 to 0.87)
1 time/day ~ <2 times/day	0.81 (0.66 to 0.997)	0.84 (0.66 to 1.07)	0.79 (0.62 to 1.01)	0.87 (0.67 to 1.14)
≥2 times/day	0.34 (0.27 to 0.42)	0.36 (0.28 to 0.46)	0.33 (0.25 to 0.42)	0.37 (0.28 to 0.49)
Green tea intake				
<6–11 times/year	reference	reference	reference	reference
6–11 times/year ~ <1 time/week	1.07 (0.87 to 1.33)	1.22 (0.95 to 1.56)	1.21 (0.93 to 1.56)	1.31 (0.99 to 1.72)
1 time/week ~ <1 time/day	0.69 (0.57 to 0.85)	1.33 (1.05 to 1.69)	1.35 (1.06 to 1.73)	1.63 (1.26 to 2.11)
≥1 time/day	1.55 (1.14 to 2.11)	2.82 (1.96 to 4.05)	3.01 (2.09 to 4.34)	3.35 (2.29 to 4.89)
Carbonated beverage intake				
<1 time/month	reference	reference	reference	reference
1 time/month ~ <1 time/week	0.37 (0.30 to 0.45)	0.999 (0.78 to 1.28)	1.10 (0.85 to 1.41)	1.10 (0.84 to 1.45)
1 time/week ~ <1 time/day	0.20 (0.15 to 0.26)	1.31 (0.94 to 1.83)	1.45 (1.02 to 2.05)	1.26 (0.88 to 1.80)
≥1 time/day	0.59 (0.25 to 1.34)	5.87 (1.74 to 19.78)	5.82 (1.89 to 17.91)	4.29 (1.32 to 13.91)

Data are presented as odds ratio (95% confidence interval).

Model 1: Adjusted for age.

Model 2: Adjusted for age, the frequency of intake of coffee, tea, and carbonated beverages.

Model 3: Adjusted for age, the frequency of intake of coffee, tea, and carbonated beverages, daily nutritional intake (total and fat), average monthly household income, education level, smoking, alcohol drinking, walking, body mass index status, and menopause status (only in women).

Table S15. Multivariable logistic regression for dyslipidemia when the frequency of intake is categorized into four groups

	Crude	Model 1	Model 2	Model 3
Men				
Coffee intake				
<1 time/week	reference	reference	reference	reference
1 time/week ~ <1 time/day	0.52 (0.39 to 0.68)	0.71 (0.52 to 0.95)	0.67 (0.49 to 0.91)	0.62 (0.46 to 0.85)
1 time/day ~ <2 times/day	1.15 (0.88 to 1.49)	0.90 (0.67 to 1.20)	0.85 (0.64 to 1.14)	0.82 (0.61 to 1.11)
≥2 times/day	1.09 (0.88 to 1.35)	0.68 (0.54 to 0.87)	0.67 (0.52 to 0.86)	0.60 (0.46 to 0.78)
Green tea intake				
<6–11 times/year	reference	reference	reference	reference
6–11 times/year ~ <1 time/week	1.28 (1.04 to 1.58)	1.40 (1.11 to 1.77)	1.36 (1.08 to 1.71)	1.37 (1.07 to 1.75)
1 time/week ~ <1 time/day	0.92 (0.77 to 1.11)	1.50 (1.22 to 1.85)	1.50 (1.21 to 1.84)	1.66 (1.34 to 2.07)
≥1 time/day	2.37 (1.85 to 3.04)	2.71 (2.03 to 3.62)	2.69 (2.02 to 3.60)	2.77 (2.07 to 3.71)
Carbonated beverage intake				
<1 time/month	reference	reference	reference	reference
1 time/month ~ <1 time/week	0.57 (0.47 to 0.68)	1.18 (0.96 to 1.46)	1.19 (0.97 to 1.46)	1.23 (0.99 to 1.53)
1 time/week ~ <1 time/day	0.32 (0.26 to 0.38)	1.17 (0.92 to 1.48)	1.15 (0.91 to 1.46)	1.19 (0.94 to 1.52)
≥1 time/day	0.19 (0.11 to 0.35)	0.97 (0.51 to 1.84)	1.02 (0.52 to 1.99)	1.10 (0.57 to 2.14)
Women				
Coffee intake				
<1 time/week	reference	reference	reference	reference
1 time/week ~ <1 time/day	0.55 (0.45 to 0.65)	0.93 (0.74 to 1.16)	0.88 (0.70 to 1.11)	0.87 (0.69 to 1.09)
1 time/day ~ <2 times/day	1.08 (0.91 to 1.27)	1.10 (0.89 to 1.37)	1.05 (0.85 to 1.30)	1.08 (0.87 to 1.35)
≥2 times/day	0.80 (0.68 to 0.94)	0.82 (0.67 to 0.997)	0.78 (0.64 to 0.96)	0.83 (0.68 to 1.02)
Green tea intake				
<6–11 times/year	reference	reference	reference	reference
6–11 times/year ~ <1 time/week	1.18 (0.99 to 1.39)	1.22 (0.9997 to 1.48)	1.21 (0.99 to 1.48)	1.28 (1.04 to 1.57)
1 time/week ~ <1 time/day	0.76 (0.65 to 0.89)	1.39 (1.15 to 1.68)	1.39 (1.15 to 1.69)	1.49 (1.23 to 1.81)
≥1 time/day	1.40 (1.09 to 1.79)	2.29 (1.67 to 3.13)	2.30 (1.69 to 3.15)	2.30 (1.67 to 3.16)
Carbonated beverage intake				
<1 time/month	reference	reference	reference	reference
1 time/month ~ <1 time/week	0.41 (0.35 to 0.47)	1.13 (0.94 to 1.36)	1.15 (0.95 to 1.38)	1.15 (0.95 to 1.40)
1 time/week ~ <1 time/day	0.18 (0.14 to 0.22)	1.01 (0.78 to 1.29)	0.9999 (0.77 to 1.29)	0.93 (0.72 to 1.21)
≥1 time/day	0.32 (0.16 to 0.63)	3.28 (1.05 to 10.25)	3.27 (1.12 to 9.53)	2.72 (1.01 to 7.35)

Data are presented as odds ratio (95% confidence interval).

Model 1: Adjusted for age.

Model 2: Adjusted for age, the frequency of intake of coffee, tea, and carbonated beverages.

Model 3: Adjusted for age, the frequency of intake of coffee, tea, and carbonated beverages, daily nutritional intake (total and fat), average monthly household income, education level, smoking, alcohol drinking, walking, body mass index status, and menopause status (only in women).

Table S16. Multivariable logistic regression for metabolic syndrome when the frequency of intake is categorized into four groups

	Crude	Model 1	Model 2	Model 3
Men				
Coffee intake				
<1 time/week	reference	reference	reference	reference
1 time/week ~ <1 time/day	0.96 (0.82 to 1.13)	1.06 (0.90 to 1.26)	1.06 (0.89 to 1.25)	1.00 (0.83 to 1.22)
1 time/day ~ <2 times/day	1.47 (1.27 to 1.72)	1.30 (1.12 to 1.52)	1.28 (1.10 to 1.50)	1.19 (0.998 to 1.42)
≥2 times/day	1.58 (1.39 to 1.79)	1.35 (1.18 to 1.54)	1.34 (1.18 to 1.53)	1.07 (0.92 to 1.24)
Green tea intake				
<6–11 times/year	reference	reference	reference	reference
6–11 times/year ~ <1 time/week	0.94 (0.84 to 1.06)	1.04 (0.92 to 1.17)	1.06 (0.94 to 1.20)	1.01 (0.88 to 1.17)
1 time/week ~ <1 time/day	0.90 (0.81 to 1.004)	1.09 (0.98 to 1.22)	1.12 (0.9996 to 1.26)	0.99 (0.86 to 1.14)
≥1 time/day	1.20 (1.03 to 1.39)	1.29 (1.11 to 1.50)	1.26 (1.09 to 1.47)	1.05 (0.88 to 1.25)
Carbonated beverage intake				
<1 time/month	reference	reference	reference	reference
1 time/month ~ <1 time/week	0.73 (0.65 to 0.81)	0.96 (0.86 to 1.08)	0.95 (0.85 to 1.07)	0.96 (0.84 to 1.09)
1 time/week ~ <1 time/day	0.58 (0.52 to 0.64)	0.99 (0.88 to 1.11)	0.97 (0.86 to 1.08)	1.01 (0.88 to 1.15)
≥1 time/day	0.49 (0.37 to 0.65)	0.97 (0.72 to 1.29)	0.94 (0.70 to 1.26)	0.93 (0.66 to 1.31)
Women				
Coffee intake				
<1 time/week	reference	reference	reference	reference
1 time/week ~ <1 time/day	0.74 (0.64 to 0.84)	1.03 (0.89 to 1.19)	1.01 (0.87 to 1.17)	1.00 (0.85 to 1.18)
1 time/day ~ <2 times/day	1.004 (0.90 to 1.13)	1.01 (0.89 to 1.14)	0.98 (0.86 to 1.12)	0.98 (0.85 to 1.14)
≥2 times/day	0.83 (0.74 to 0.93)	0.94 (0.83 to 1.05)	0.91 (0.81 to 1.03)	0.85 (0.74 to 0.97)
Green tea intake				
<6–11 times/year	reference	reference	reference	reference
6–11 times/year ~ <1 time/week	0.85 (0.76 to 0.96)	0.99 (0.88 to 1.12)	0.98 (0.86 to 1.10)	1.01 (0.88 to 1.16)
1 time/week ~ <1 time/day	0.64 (0.58 to 0.72)	0.93 (0.83 to 1.05)	0.92 (0.82 to 1.03)	0.98 (0.86 to 1.12)
≥1 time/day	0.88 (0.75 to 1.03)	1.15 (0.97 to 1.36)	1.15 (0.97 to 1.36)	1.15 (0.95 to 1.39)
Carbonated beverage intake				
<1 time/month	reference	reference	reference	reference
1 time/month ~ <1 time/week	0.71 (0.64 to 0.78)	1.23 (1.11 to 1.37)	1.24 (1.11 to 1.38)	1.19 (1.05 to 1.34)
1 time/week ~ <1 time/day	0.50 (0.44 to 0.56)	1.42 (1.24 to 1.61)	1.42 (1.25 to 1.62)	1.26 (1.09 to 1.46)
≥1 time/day	0.57 (0.37 to 0.86)	1.96 (1.22 to 3.15)	1.94 (1.20 to 3.12)	2.23 (1.43 to 3.49)

Data are presented as odds ratio (95% confidence interval).

Model 1: Adjusted for age.

Model 2: Adjusted for age, the frequency of intake of coffee, tea, and carbonated beverages.

Model 3: Adjusted for age, the frequency of intake of coffee, tea, and carbonated beverages, daily nutritional intake (total and fat), average monthly household income, education level, smoking, alcohol drinking, walking, body mass index status, and menopause status (only in women).
